# Supplementation of Vitamin K1 in Dogs With Chronic Enteropathy

**DOI:** 10.1111/jvim.70111

**Published:** 2025-05-02

**Authors:** Jillian Myers Smith, Christopher Keenan Smith, Xiaojuan Zhu, Ashley Hartley, Elizabeth M. Lennon

**Affiliations:** ^1^ Small Animal Clinical Sciences University of Tennessee Knoxville Tennessee USA; ^2^ Office of Innovative Technology University of Tennessee Knoxville Tennessee USA; ^3^ Department of Small Animal Clinical Science University of Pennsylvania School of Veterinary Medicine Philadelphia Pennsylvania USA

**Keywords:** canine, gastrointestinal, malabsorption, phytonadione

## Abstract

**Background:**

Information regarding measurement and supplementation of vitamin K1 (vitK1) in dogs with chronic enteropathy (CE) is limited.

**Hypothesis/Objectives:**

Compare vitK1 concentrations of healthy dogs to dogs with CE and determine if supplementation with vitK1 increases vitK1 concentrations compared to placebo.

**Animals:**

Twenty client‐owned dogs with CE and 20 healthy university‐owned research colony dogs.

**Methods:**

Prospective, randomized, placebo‐controlled study. Dogs with CE were randomly assigned to receive placebo or vitk1 2.5 mg/kg PO q12h for 3 weeks. Vitamin K concentrations were measured pre‐ and post supplementation using liquid chromatography tandem mass spectrometry and compared to vitK1 concentrations in the healthy cohort.

**Results:**

All healthy dogs had initial vitK1 median concentrations of 0.10 ng/mL (interquartile range [IQR], 0.05), which was similar to dogs that received either placebo (*n* = 5; 0.10 ng/mL; IQR, 0.05) or vitK1 (*n* = 7; 0.10 ng/mL; IQR, 0.05) before supplementation. Dogs with CE receiving vitK1 had increased vitK1 concentrations (12.5 ng/mL; IQR, 4.1) after 3 weeks of supplementation compared with baseline (0.10 ng/mL; *p* < 0.001), placebo group after 3 weeks (0.10 ng/mL; *p* < 0.0001) and healthy dogs (0.10 ng/mL; *p* < 0.004).

**Conclusions and Clinical Importance:**

Oral supplementation with vitK1 increased vitK1 concentration in the serum of dogs with CE, but a clinical benefit from increased vitK1 concentrations was not identified. The absence of difference in vitK1 concentrations between healthy and CE dogs before supplementation requires additional investigation.

**Abbreviations**
AAFCO: Association of American Feed Control Officials
CE: chronic enteropathy
CEP: dogs with chronic enteropathy supplemented with a placebo
CEK: dogs with chronic enteropathy supplemented with vitamin K1
CIBDAI: canine inflammatory bowel disease activity index
CKD: chronic kidney disease
CRP: c‐reactive protein
GI: gastrointestinal
Glu‐ OC: undercarboxylated osteocalcin enzyme immunoassay
Gla‐ OC: carboxylated osteocalcin enzyme immunoassay
IACUC: Institutional Animal Care and Use Committee
IBD: inflammatory bowel disease
IRIS: International Renal Interest Society
ISO: International Organization for Standardization
LC/MS/MS: liquid chromatography tandem mass spectrometry
PLE: protein losing enteropathy
PT: prothrombin time
ucOC: undercarboxylated osteocalcin
%ucOC: percent undercarboxylated osteocalcin
VitK1: vitamin K1

## 1 Introduction

Chronic enteropathy (CE) in dogs encompasses a variety of persistent, debilitating intestinal diseases that can result in intestinal barrier dysfunction, altered intestinal immunity, and nutrient malabsorption. For a diagnosis of CE, gastrointestinal (GI) signs (e.g., vomiting, diarrhea, regurgitation, flatulence) must be present for > 3 weeks [1, 2], and other potential causes (e.g., parasites, neoplasia, endocrinopathy) must be ruled out [3]. Further refinement of the diagnosis may be possible with diet trials or immunosuppressive treatments (food‐responsive and immunosuppressant‐responsive enteropathies, respectively) [4]. In both humans and dogs with chronic intestinal diseases such as CE and inflammatory bowel disease (IBD), marked decreases in many essential nutrients may occur [5], including cobalamin [6], amino acids [7], and magnesium [8]. If CE is associated with protein‐losing enteropathy (PLE), the loss of proteins, lymphocytes, and lipids can be clinically relevant and life‐threatening [9]. Malabsorption of fat raises concern for the development of deficiencies in fat‐soluble vitamins, such as vitamins D [10] (demonstrated in dogs) and K [11, 12] (demonstrated in humans).

Accurate determination of vitamin K1 (vitK1) status is challenging. Direct measurement of vitK1 is analytically difficult and often not practical, because it is rapidly used and not stored in large amounts in the body [13]. Although vitK1 can be measured in humans, it is markedly affected by diet, meal pattern, the presence of triglycerides, and it requires specialized laboratories and equipment for optimization because of extremely low circulating concentrations [14]. Because of these limitations, measurement of vitK1 functional activity is now considered to be a more accurate reflection of vitK1 status in humans [15, 16]. In people, functional activity can be assessed by determining the percentage of undercarboxylated osteocalcin (%ucOC), which is a sensitive functional marker of vitK1 [17]. Unfortunately, no validated tests for %ucOC in dogs currently are available. Thus, direct measurement is achieved using liquid chromatography tandem mass spectrometry (LC/MS/MS) [18]. The normal range of serum vitK1 concentration in dogs is currently unknown.

Our primary aim was to compare directly measured vitK1 concentrations in healthy dogs and dogs with CE using LC/MS/MS. A secondary aim was to investigate whether supplementation of vitK1 in dogs with CE would significantly increase vitK1 concentrations. We hypothesized that dogs with CE would have lower concentrations of vitK1 compared with healthy dogs, and that supplementation with vitK1 would increase measurable vitK1 concentrations.

## 2 Materials and Methods

### 2.1 Animals

Ours was a prospective, randomized, placebo‐controlled study, which was approved by the University of Tennessee Institutional Animal Care and Use Committee (IACUC; protocol: 2701–0619). Two groups of dogs were evaluated in the study. The first group consisted of 20 client‐owned dogs with CE that were referred to the veterinary teaching hospital and prospectively enrolled in accordance with the study's inclusion and exclusion criteria. Inclusion criteria consisted of having one or more of the following clinical signs: vomiting, diarrhea, constipation, regurgitation, flatulence, or weight loss for at least 3 weeks with no other identifiable systemic or nonintestinal cause based on CBC, serum biochemistry profile, imaging, and additional screening (see below) as deemed appropriate by the attending clinician. Broad‐spectrum deworming (with or without negative fecal sample test results) and abdominal imaging (radiography and ultrasound examination interpreted by a board‐certified veterinary radiologist) were performed at study enrollment or before (within 3 months) enrollment. Patients diagnosed with CE based on the aforementioned criteria were considered eligible. Intestinal biopsies were not required in accordance with current standards of diagnosing CE [1]. Patients were excluded if they had a concurrent condition known to affect vitK1 status, osteocalcin concentrations, or GI health, including severe liver disease or failure [19], cholestasis [20], clinically relevant proteinuria (urine protein: creatinine ratio > 2.0), or moderate to severe chronic kidney disease (CKD), defined as International Renal Interest Society (IRIS) stage III or higher [21], hyperadrenocorticism [22], recent and prolonged (> 2 weeks) use of PO antibiotics before enrollment [23], severe pancreatitis [24], hypoadrenocorticism, systemic or bone neoplasia [25], or other clinically relevant bone pathology [26] (defined as bone or joint infection or recent orthopedic surgical procedure). The second group evaluated was a cohort of 20 healthy university‐owned research colony dogs that were cared for according to the university's IACUC standard protocol.

### 2.2 Dogs With CE

Owners willing to have their dogs enrolled in the study signed an informed consent acknowledgment, and each owner confirmed the dog had been fasted for 12 h before any blood collection. For each dog with CE, samples (blood, urine, feces) were collected at the initial visit (visit 1). In accordance with the dog's weight (not to exceed 1% of body weight), blood was collected from the jugular vein for routine analysis, consisting of CBC, serum biochemistry profile, C‐reactive protein (CRP) concentration, and prothrombin time (PT).

The 20 enrolled dogs with CE were randomly assigned using www.random.org to one of two groups: placebo group (CEP) or a vitK1 supplementation group (CEK). Each dog enrolled in the CEP group received 0.25 mL of sterile saline by SC injection at enrollment, followed by a daily placebo pill (inert gelatin lactose capsule formulated by the university's pharmacy) given PO once daily. For dogs assigned to the CEK group, a 2.5 mg/kg SC injection of 10 mg/mL phytonadione (VetOne, Boise, ID) was administered upon study enrollment, and a prescription of a PO phytonadione supplement (Vedco, St. Joseph, MO) was prescribed at 2.5 mg/kg PO q12h, rounded to the nearest capsule availability (25 or 50 mg capsules of vitK1). Each treatment (placebo or vitK1 supplement) was prescribed for a course of 21 days. Owners were blinded to the group their dog was assigned to, but the person administering the injection was not blinded. Additional treatments for CE were at the discretion of the attending clinician (e.g., diet changes, immunosuppressive drugs).

All dogs with CE were returned 3 weeks later (visit 2) to re‐evaluate blood test results (serum biochemistry profile with electrolytes) and to have samples collected for the study. Additional laboratory testing was performed at the discretion of the attending veterinarian. Each owner was asked to complete a questionnaire to classify the dog under the Canine Inflammatory Bowel Disease Activity Index (CIBDAI) [27] at both visits.

### 2.3 Healthy Dogs

Twenty research colony dogs receiving an Association of American Feed Control Officials (AAFCO) certified commercial adult maintenance dog food (Purina One Lamb and Rice, Purina, Neenah, WI) were used as the healthy control population to provide samples for direct vitK1 measurement in a healthy canine population. All dogs were beagle/hound mixes between 3 and 4 years of age and weighed on average 5.4 kg (range, 4–11.1 kg) and were free of any clinical signs of GI disease or other exclusionary criteria. The dogs were fasted for 12 h before sample collection, and collection volume was based on individual weight per IACUC standards.

### 2.4 Sample Analysis

Blood was collected into red top tubes, allowed to clot for 30 min, and then centrifuged at 3000 rpm for 15 min on a LMC‐3000 centrifuge (Grant Instruments LLC, Cambridgeshire, UK). Serum then was transferred into cryovials in 500 μL aliquots for routine analyses in the university's clinical pathology laboratory, or 250 μL aliquots for tests that were not performed in‐hospital (direct vitK1 measurement). Aliquots were kept on ice and transported to a −80°C freezer within 1 h.

For PT evaluation, the citrated sample was submitted to the university laboratory within 10 min of collection and analyzed using a benchtop coagulation analyzer (Stago compact instrument, Stago, Parsippany, NJ, USA). Measurement of CRP was performed using an automated turbidimetric immunoassay that utilizes canine‐specific reagent antibodies (Canine CRP Assay, Gentian Diagnostics AS, Norway). In clinically healthy dogs, CRP concentration has been documented to be < 10 mg/L [28]. Samples for cobalamin analysis were submitted to the Texas A&M University GI laboratory (College Station, TX) for measurement.

For direct vitK1 measurement, samples were submitted through Heartland Assays (Ames, IA). The laboratory is International Organization for Standardization (ISO 17025) certified and participates in external quality assurance and proficiency testing for vitK1 and vitK2 according to the Vitamin K External Quality Assurance Scheme. A previously validated technique (LC/MS/MS) was employed to measure direct vitK1 [14].

### 2.5 Statistical Analysis

A postexperimental power analysis was performed and 18 dogs per treatment group (total 36 dogs) were required to have 80% power using a two‐sample *t* test to detect a significant difference between two treatment groups with a mean difference (μ1—μ2) of 4.82 and SD of 5. (PASS 2024, version 24.0.2). Descriptive statistics were performed for the following: age, weight, PT, albumin, cholesterol, CRP, CIBDAI, and vitK1. Each response variable was analyzed for normality using the Shapiro–Wilk test and Q–Q plots. Both CRP and vitK1 violated the assumption of normality, but the other variables were found to be normally distributed.

Because of the limited sample size, a Mann–Whitney U test was used to compare the difference between treatment groups in age and weight. Fisher's exact test was used to test the association between the treatment groups and sex. Two‐way repeated measures analysis of variance (ANOVA) was performed on the normal chemistry measurements to determine whether a significant difference existed between the treatment groups, and between two visits, and also the effect of the treatment by visit interaction. Two‐way repeated measures ANOVA on ranks was performed on CRP and vitK1 to detect whether there was a significant effect of treatments, visits, and the treatment by visit interaction. Least square means were computed and separated by Fisher's least significant difference (LSD). All statistical assumptions regarding normality and equality of variances were met. A Wilcoxon signed rank test was used to compare the dogs with CE to the healthy population because all 20 healthy dogs had a direct vitK1 measurement of 0.10 ng/mL. *p* < 0.05 was considered significant. SAS, version 9.4, release TS1M8, was used for all analyses. (SAS Institute Inc., Cary, NC, USA.)

## 3 Results

### 3.1 Animals: Enrollment, Signalment, Diagnosis, and Treatments

Twenty‐two client‐owned dogs diagnosed with CE were enrolled. Twenty dogs met inclusion criteria because one dog died before finishing the study, and another dog was not returned for follow‐up appointments. There was no difference in age (*p* = 0.82), weight (*p* = 0.85), or sex (*p* = 1.00) between the CEK and CEP groups. Underlying comorbidities regardless of CEK or CEP grouping included hypothyroidism (1), chronic urinary infections (2), urinary calculi (2), IRIS stage I CKD (2), brachycephalic obstructive airway syndrome (2), anemia (1), heart murmur (3), osteoarthritis (2), diabetes mellitus (1), periodontal disease (1), mixed hepatopathy (1), and goniodysgenesis (1). A summary of demographic information and additional treatments the dogs received is presented in Table 1.

**TABLE 1:** Clinical and treatment data for all study groups.

Group	Years of age median (range)	Weight kg median (range)	Sex	Breed	Diet changes	Additional therapy
CE + Vit K	9 (2–14)	8.15 (4–27)	6 MN	Beagle (1)	Hill's GI biome (1)	Calcitriol (1)
			4 FS	Cairn Terrier (1)	Homemade (3)	Clopidegrel (2)
				Cocker Spaniel (1)	Purina HA feline (3)	Fenbendazole (6)
				German Shephard Dog (1)	Purina salmon (1)	Probiotic (4)
				Maltese (1)	Royal canin hydrolyzed protein (2)	Corticosteroids (6)
				Mixed breed (1)		
				Standard Poodle (1)		
				Yorkshire Terrier (3)		
CE + Placebo	7 (4–15)	10.6 (3.5–28)	6 MN	Cocker Spaniel (1)	Purina HA (6)	Capromorelin (1)
			4 FS	Dachshund (1)	Purina HA feline (1)	Cobalamin (2)
				English Bulldog (1)	Royal canin GI low Fat (1)	Clopidegrel (3)
				French Bulldog (1)	Royal canin rabbit & potato (1)	Fenbendazole (3)
				Goldendoodle (1)		Mirtazapine (1)
				Maltese (1)	No change (1)	Probiotic (6)
				Miniature Schnauzer (1)		Corticosteroids (3)
				Mixed breed (1)		
				Standard Poodle (1)		
				Yorkshire Terrier (1)		

Clinical signs of dogs in the CEK group at visit 1 were as follows: 8/10 dogs had diarrhea, 5/10 had vomiting, 3/10 had weight loss, 3/10 had effusion (either thoracic or abdominal), and 1/10 was reported to have a decreased appetite, 1/10 was reported to have chronic constipation, and 1/10 had tenesmus and hematochezia. After supplementation (visit 2), diarrhea was reported in 4/10, vomiting in 1/10, weight loss in 2/10, effusion in 1/10, decreased appetite in 1/10, and 1/10 each had constipation and tenesmus with hematochezia. The clinical signs of the dogs in the CEP group at visit 1 were as follows: 8/10 had diarrhea, 3/10 had vomiting, 2/10 had weight loss, 3/10 had effusion, and 0/10 had decreased appetite. At visit 2, 5/10 had diarrhea, 2/10 had vomiting, 3/10 had weight loss, 1/10 had effusion, and 0/10 had decreased appetite.

### 3.2 Follow‐up Testing

The average time between blood collections (visits 1 and 2) for the CEK group was 22 days (range, 16–26 days) and 21 days (range, 15–29 days) for the CEP group. These times were not significantly different.

### 3.3 Selected Biochemistry Results and Ancillary Tests

Serum albumin concentrations increased significantly at visit 2 independent of treatment group. Similarly, serum cholesterol concentrations increased significantly at visit 2, independent of treatment group (Table 2).

**TABLE 2:** Selected clinicopathologic data between different treatment groups and visits 1 and 2; this does not include the healthy control cohort.

Test	Treatment	Visits	Mean (SD) or ^a^Median (IQR)
PT (6.8–8.7 s) (*n* = 11)	CEK (*n* = 7)	1	7.7 (0.5)
		2	7.9 (0.6)
	CEP (*n* = 4)	1	8.5 (0.4)
		2	8.1 (0.6)
Albumin (3.2–4.3 g/dL)^b^ (*n* = 20)	CEK (*n* = 10)	1	2.5 (0.9)
		2	2.9 (0.7)
	CEP (*n* = 10)	1	2.1 (0.9)
		2	2.7 (0.7)
Globulin (1.9–3.1 g/dL) (*n* = 20)	CEK (*n* = 10)	1	2.6 (0.9)
		2	2.6 (0.6)
	CEP (*n* = 10)	1	2.0 (0.8)
		2	2.1 (0.3)
^b^Cholesterol (130–354 mg/dL) (*n* = 20)	CEK (*n* = 10)	1	132 (55)
		2	169 (89)
	CEP (*n* = 10)	1	130 (59)
		2	159 (69)
Total calcium (10–12 mg/dL) (*n* = 20)	CEK (*n* = 10)	1	9.0 (1.6)
		2	9.6 (1.4)
	CEP (*n* = 10)	1	8.5 (2.0)
		2	8.9 (2.1)
^a^, ^b^C reactive protein (< 10 mg/L) (*n* = 20)	CEK (*n* = 10)	1	3 (IQR 7)
		2	3 (IQR 0)
	CEP (*n* = 10)	1	8 (IQR 6)
		2	3 (IQR 0)
^a^, ^b^Direct vitK1 in ng/mL (*n* = 12)	CEK (*n* = 7)	1	0.10 (IQR 0.1)
		2	12.50 (IQR 4.1)
	CEP (*n* = 5)	1	0.10 (IQR 0)
		2	0.10 (IQR 0.1)

*Note:* Data presented as mean and SD for parametric data or median and interquartile range (IQR) for nonparametric data for CE dogs in either vitK or placebo groups at visits 1 or 2. Reference intervals are listed for each test.

Abbreviations: CEK, dogs with CE supplemented with vitk1; CEP, dogs with CE given placebo; PT, prothrombin time; vitK1, vitamin K1.

^a^ Value is significantly different from placebo at the same visit.

^b^ Value is significantly different than visit 1 with a *p* < 0.05.

Serum cobalamin concentration was measured in 14/20 dogs with CE pre‐supplementation. The mean cobalamin concentration was 368 ± 256 ng/L. Nine of 14 of these dogs were hypocobalaminemic and were supplemented with cobalamin (guidelines outlined by Texas A&M [29]) based on recommendations from their attending clinicians. Histoplasmosis testing (urine antigen enzyme immunoassay [EIA]; MiraVista Labs, Indianapolis, IN) was performed in half of the enrolled dogs (5/10 in both the CEP and CEK groups); all results were negative. All dogs had either a documented negative fecal flotation or were dewormed with a broad‐spectrum dewormer (fenbendazole, Merck & Co. Inc., Rahway, NJ, USA), or both. Selected clinicopathologic data are included in Table 2.

### 3.4 CIBDAI, PT, and CRP

All 20 dogs with CE had CIBDAI scores evaluated at visits 1 and 2. No differences in scores between the CEK and CEP groups were found at visit 1 or visit 2. The treatment by visit interaction also was not significant. A significant difference was noted between visits; CIBDAI scores were lower at visit 2 compared with visit 1 (*p* = 0.003) in both groups, but not different between CEP and CEK groups at either visit. The average CIBDAI score in the CEK group at visit 1 was 7 ± 3.7 and postsupplementation was 4 ± 3.3. The average CIBDAI score for the CEP group at visit 1 was 6 ± 2.3) and at visit 2 was 4 ± 3.2).

Eleven dogs with CE had PT measured pre‐ and post‐treatment (seven CEK, four CEP) and all results were within the normal reference range (Table 2).

All 20 dogs had pre‐ and post‐treatment CRP measured, and a significant difference was found between visits 1 and 2 regardless of treatment group (Table 2).

### 3.5 Direct Vitamin K

**FIGURE 1:**
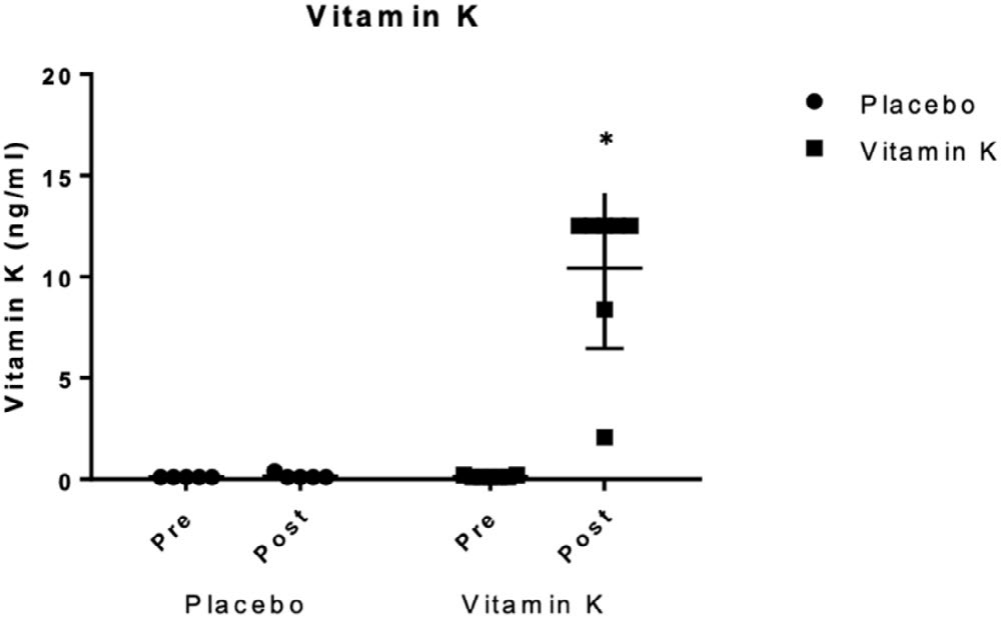
Comparison of individual data of direct vitK1 (presented in ng/mL) between 12 dogs with chronic enteropathy supplemented with placebo versus vitK1 at visit 1 (pre) and visit 2 (post). The single horizontal line represents the median (12.5 ng/mL (IQR 4.1). Squares or circles indicate individual data points. *p < 0.0001.

In total, pre‐ and post‐supplementation samples from 12 dogs with CE were submitted for direct serum vitK1 measurement (seven CEK dogs and five CEP dogs). The median concentration of vitK1 in the CEK group at visits 1 and 2 was 0.1 ng/mL (IQR, 0.05; range, 0.1–0.2) and 12.5 ng/mL (IQR 4.1; range, 2.5–12.5), respectively. Significant treatment (*p* < 0.001), visit (*p* < 0.001), and treatment by visit (*p* = 0.01) effects were observed in vitK1 concentrations (Figure 1). The CEK group at visit 2 was higher than the CEK group at visit 1 and higher than the CEP group at both visits (*p* < 0.001). No difference was found in the CEP group between visit 1 and visit 2 (Table 2).

Twenty healthy dogs had a direct vitK1 measurement of 0.10 ng/mL, which was the lowest concentration able to be reported by the laboratory. When comparing the healthy population to the dogs with CE, no difference in direct vitK1 was found between CEP dogs at either visit or CEK dogs at visit 1 and healthy dogs (*p* = 0.50). However, a significant difference was found between CEK dogs at visit 2 and the healthy dogs (*p* = 0.004).

## 4 Discussion

Although several studies have examined the concentrations of different vitamins in dogs with CE [30], to our knowledge, vitK1 concentrations in dogs with CE and with reference to normal vitK1 concentrations in a healthy cohort have not been reported previously. Although our study showed no difference in vitK1 concentrations between dogs with CE and healthy dogs at baseline (visit 1), a significant increase in vitK1 concentrations in dogs with CE was identified after supplementation. Supplementing dogs with a 2.5 mg/kg SC injection of phytonadione followed by phytonadione supplementation prescribed at 2.5 mg/kg PO q12h for 21 days substantially increased serum vitK1 concentrations.

Fat‐soluble vitamin deficiencies in patients with CE and in patients with other forms of GI disease sometimes can lead to exacerbated inflammation and worse outcomes. A previous study concluded that a higher vitK1 concentration was associated with lower concentrations of inflammatory markers in people [31]. In children suffering from IBD, vitK1 deficiency has been documented in up to 54% of cases, and those pediatric patients with lower vitK1 concentrations were documented to have worse disease activity index scores [32]. Adults with IBD also have been documented to be deficient in vitK1, which may be related to decreased dietary fat intake and impaired intestinal absorptive capabilities secondary to inflammation [33]. In our study, the measured marker and index for inflammation (CRP and CIBDAI, respectively) improved between visits 1 and 2, regardless of the treatment group. Given a lack of evidence that an increase in vitK1 concentration affected these markers, conclusions cannot be made regarding the effect vitK1 has on decreasing inflammation. This finding was similar to the effects seen in serum albumin and cholesterol concentrations, which improved over time but were independent of treatment group. The various treatments provided to the dogs were not standardized (e.g., diet alterations, corticosteroid treatment, probiotics), and these likely contributed to the improvements seen in CIBDAI [34, 35, 36], as well as CRP, albumin and cholesterol concentrations.

Historically, PT has been used to assess vitK1 status [37]. Correlating low vitK1 concentration with prolonged PT or proteins induced by vitamin K absence (PIVKA‐II) is not a sensitive or specific way to determine vitK1 concentrations in the body [15]. Additionally, PIVKA‐II is not readily available through veterinary laboratories, and PIVKA‐II is a retrospective indicator of vitK1 status [38]. In our study, PT was evaluated in 11 dogs with CE and showed no significant changes regardless of visit or treatment group. Currently, the extent of decrease in vitk1 concentration necessary to affect PT is unknown and warrants further investigation. Regardless, when considering the lack of clinical signs (e.g., hemorrhage, ecchymoses, PT prolongation) of vitK1 deficiency, it is impossible to know if increasing the dogs' vitK1 concentration had a measurable impact.

Serum vitk1 concentrations in dogs are not routinely measured, nor are established reference intervals available. The results presented here document reliable measurement of serum vitK1 using LC/MS/MS. In our sample of healthy dogs, all reported concentrations of vitK1 were < 0.1 ng/mL, which was the lowest quantified measurement attainable by the laboratory. This result was not different from that of vitK1 found in either group of dogs with CE at visit 1 or CEP dogs at visit 2. There are several potential reasons why vitK1 concentrations were not different between healthy dogs and dogs with CE at the aforementioned time points, with the most plausible being that the lowest concentrations quantifiable by LC/MS/MS are above the actual difference between the groups. That is, LC/MS/MS may not be the most sensitive method for vitK1 detection. This possibility could support investigation of percent undercarboxylated osteocalcin (%ucOC) as a more sensitive method for identifying vitK1 deficiency, as is done in humans [39], which theoretically may identify measurable vitK differences between healthy dogs and dogs with CE. Another possible reason for the lack of difference could be that the CE in our sample population was not severe enough to cause a measurable difference in vitK1 concentrations. Finally, it is possible that the decrease in vitK1 concentrations seen in certain chronic GI diseases in humans is not clinically manifested in dogs with CE.

Serum vitK1 concentrations significantly increased in dogs with CE using the protocol in our study, without adverse effects. This dosing protocol is complementary to other studies that have described dosing of vitK1. A previous study, for instance, established the IV vitK1 requirement to preserve PT time after cholecystonephrostomy in dogs [40]. Another study evaluated the amount of vitK1 needed to normalize PT after the use of warfarin and found that a dosage of 2.2 mg/kg of vitK1 could be given PO for 3 days [41]. Direct vitK1 concentrations were not measured in either study, and longer courses of treatment were not evaluated.

Our study had some limitations. The major limitations included the small number of dogs enrolled and the lack of standardization of treatments for CE, which causes difficulty in interpreting the benefits of vitK1 treatment alone. Improvements in some of the clinical markers evaluated (e.g., albumin, CIBDAI scores, cholesterol) may be the result of a combination of vitK1 supplementation and the animal's other prescribed treatments. Another limitation was measuring healthy control dog vitK1 concentration at only one time point. Normal variations may occur in vitK concentrations, and including additional measurements would have been useful to investigate for such deviations. As stated previously, the lowest limit of detection of vitK1 using LC/MS/MS was 0.1 ng/mL, which was the result obtained for CEP dogs as well as healthy dogs. Thus, this method of measuring vitK1 may not be sensitive enough to detect differences.

Future studies should focus on investigating %ucOC as a measure of functional vitK in dogs, and its efficacy and relationship with other forms of vitK such as vitK2 and vitK3. Another area of interest to investigate would be to determine if vitK1 can act exclusively as a mediator of inflammation in patients with CE (or other disease) and potentially use LC/MS/MS and %ucOC to provide evidence. Based on our study's limitations, future studies should use standardized treatment protocols in all groups in conjunction with vitK1 supplementation.

## 5 Conclusion

In conclusion, we did not find a difference in serum vitamin K concentrations between healthy dogs and dogs with CE (before supplementation) using LC/MS/MS, and failure to find a difference may be a result of inadequate sensitivity to detect a difference using LC/MS/MS. Supplementing dogs with a 2.5 mg/kg SC injection of phytonadione followed by PO phytonadione supplementation prescribed at 2.5 mg/kg PO q12h can reliably increase serum vitK concentrations in dogs with CE without any observable adverse effects.

## Disclosure

Authors declare no off‐label use of antimicrobials.

## Ethics Statement

Institutional Animal Care and Use Committee approval was granted for this study by the University of Tennessee, Protocol Number: 2701–0619. Authors declare that human ethics approval was not needed.

## Conflicts of Interest

The authors declare no conflicts of interest.
